# The mediating effect of burnout on the relationship between workplace culture and intent to stay among registered nurses in Saudi Arabia

**DOI:** 10.1371/journal.pone.0352383

**Published:** 2026-06-25

**Authors:** Mesheil Alalyani, Joyce Buta, Benito Areola Jr, Ohoud Naif Aldughmi, Ingrid Jacinto-Caspillo, Sumathi Robert Shanmugam, Richard Maestrado, Allen Joshua Dominguez, Analita Gonzales, Nuha Ayad H. Alatawi, Romeo Mostoles, Reem Humaidi Alalawi, Eddieson Pasay an

**Affiliations:** 1 Department of Adult and Advanced Nursing Care, College of Nursing, King Khalid University, Khamis, Saudi Arabia; 2 Department of Maternal and Child Nursing, College of Nursing, Qassim University, Qassim City, Saudi Arabia; 3 Department of Nursing Education, College of Nursing, Qassim University, Qassim City, Saudi Arabia; 4 Medical and Surgical Nursing Department, College of Nursing, Northern Border University, Arar, Kingdom of Saudi Arabia; 5 College of Nursing, Department of Maternity and Pediatric Nursing, Princess Nourah bint Abdulrahman University, Riyadh City, Kingdom of Saudi Arabia; 6 Medical-Surgical Nursing Department, College of Nursing, University of Hail, Hail City, Saudi Arabia; 7 Department of Nursing Education, Shaqra University, Riyadh, Saudi Arabia; 8 Nursing Administration and Education Department, Faculty of Nursing, University of Tabuk, Kingdom of Saudi Arabia; 9 Assistant Professor, Medical-Surgical Department, Faculty of Nursing, University of Tabuk, Kingdom of Saudi Arabia; 10 Department of Mental Health, College of Nursing, University of Hail, Hail City, Saudi Arabia; 11 Department of Nursing Administration and Education, King Khalid University, Abha, Saudi Arabia; University of Hafr Al-Batin, SAUDI ARABIA

## Abstract

**Background:**

Nurse turnover is a critical challenge to healthcare quality in Saudi Arabia, yet the mechanisms linking workplace culture to retention intentions remain unclear. While workplace culture and burnout have each been associated with turnover intentions, no study in the Saudi nursing context has examined whether burnout operates as an intermediary mechanism through which workplace culture influences nurses’ decisions to stay. Grounded in the Job Demands-Resources model, this study tested whether burnout mediates the relationship between workplace culture and intent to stay among registered nurses in Saudi Arabia.

**Methods:**

A cross-sectional, correlational design was employed. The sample comprised 355 full-time registered nurses from five Ministry of Health hospitals in the Hail and Qassim regions of Saudi Arabia. Nurses were selected using proportionate stratified random sampling, with geographic region and hospital as stratification factors. The population of 2,500 nurses was divided into two regional strata; 240 nurses were randomly selected from Hail (three hospitals) and 160 from Qassim (two hospitals), proportional to the nurse population in each region. Data were collected via self-administered online surveys (Google Forms) from November 15, 2025, to January 15, 2026. Workplace culture was measured with the Organizational Culture Survey, burnout with the Copenhagen Burnout Inventory, and intent to stay with the Intent to Stay Scale. Structural equation modeling with bootstrapping (5,000 samples) was used to test the mediation hypothesis.

**Results:**

Participants reported moderate perceptions of workplace culture (M = 2.88, SD = 0.73), high burnout (M = 3.58, SD = 0.65), and low intent to stay (M = 2.66, SD = 0.67). Workplace culture was negatively correlated with burnout (r = −0.34, p < .001) and positively correlated with intent to stay (r = 0.57, p < .001). Burnout was negatively correlated with intent to stay (r = −0.43, p < .001). In the SEM, workplace culture had a significant direct effect on intent to stay (β = 0.48, p < .001). The indirect effect of workplace culture on intent to stay through burnout was significant (β = 0.09, 95% CI [0.05, 0.16], p < .001), indicating partial mediation. The model explained 11.6% of the variance in burnout and 38.9% of the variance in intent to stay. Model fit was mixed: RMSEA and SRMR indicated good fit, while CFI and TLI were marginal (χ²(412) = 847.32, χ²/df = 2.06, CFI = 0.94, TLI = 0.93, RMSEA = 0.055 [90% CI 0.050, 0.060], SRMR = 0.048).

**Conclusion:**

In this cross-sectional study of Saudi nurses, workplace culture was associated with intent to stay through both a direct association and an indirect association via burnout. These findings are consistent with the Job Demands-Resources model and suggest that workplace culture and burnout are co-occurring factors related to retention attitudes.

## Introduction

Saudi Arabia’s health care delivery system is currently undergoing rapid development in response to increasing demand for health care driven by Vision 2030. Over the next decade growth in population will result in an increase in both quality and quantity of health care needs throughout the kingdom. This expected growth will result in a number of significant governmental initiatives that are being planned to promote an increase in the number of healthcare professionals employed in health service organizations who are Saudi nationals [1]. These factors will only add to the current stress on the existing healthcare delivery system. One major challenge facing the current healthcare delivery system in Saudi Arabia is having a stable workforce of nurses. High turnover rates amongst nurses has become one of the largest threats to quality of care and organizational viability [2,3]. Due to demographic characteristics of the nursing workforce in Saudi Arabia it is especially vulnerable to pressures relating to retention. The majority of the nursing workforce in Saudi Arabia consists of young women who are predominantly Saudi national. They are expected to provide highly complex tertiary care services within evolving environments [4,5]. Understanding what mechanisms (organizational and psychological) determine whether nurses decide to remain at their position or not is critical to developing evidence-based strategies designed to reduce or prevent nurse turnover in Saudi Arabia.

Work environment culture is one of several key organizational factors that determine how employees view their work environment [6]. Work environment culture refers to the shared values, norms and practices of professionals interacting with each other, communicating with each other, and engaging with organizational support systems [6,7]. Research conducted internationally suggests that positive work environment cultures characterized by collaborative decision-making processes, supportive leadership styles and open communication processes are associated with greater job satisfaction and lower turnover intentions among nurses [6–9]. Conversely rigid or unsuitable work environment cultures have been found to predict withdrawal behaviors including voluntary exit [10]. In studies conducted in Saudi Arabia associations between organizational support and job satisfaction have been identified [11] but not yet studied were the specific pathways through which work environment culture affects retention.

Burnout is likely another factor influencing retention. Burnout has been conceptualized as a syndrome composed of emotional exhaustion, depersonalization, and decreased personal accomplishments resulting from prolonged exposure to stressors in the workplace [12]. Burnout also has been identified as a major cause of nurse turnover in healthcare systems around the world [13–15]. Nurses in Saudi Arabia have reported high levels of burnout due to excessive workloads, the emotional demands of patient care and limited availability of needed resources [4,11]. The Copenhagen Burnout Inventory (CBI) examines burnout through three subscales: personal burnout; work-related burnout; and client-related burnout. It assesses burnout based on both organizational/interpersonal aspects [16]. Even though burnout has been shown to be strongly correlated with an individual’s intention to leave [17,18]; its role as an intermediate variable connecting work environment culture and retention versus simply being another correlating factor is unknown in the nursing literature in Saudi Arabia.

The JD-R Model [19] was used to identify the interaction among these factors. The JD-R Model indicates that job resources (e.g., workplace culture that supports employees, organizational support, autonomy), can reduce the impact of job demands (e.g., high workloads, emotionally demanding work) on worker strain. In addition, when job resources are sufficient, workers experience decreased levels of exhaustion and increased levels of engagement. Engagement is positively related to retention [19,20]. Therefore, based upon the JD-R Model, workplace culture is one type of job resource that functions as a context for reducing burnout through providing social support, role membership participation, and creating a sense of psychological safety. On the flip side, burnout represents the strain mechanism that is associated with a lack of sufficient job resources leading to withdrawal cognitions like an intent to leave. Based upon the JD-R Model, therefore, workplace culture can be expected to affect a worker’s decision to remain at their organization via two paths: a direct path (e.g., increasing job satisfaction and organizational commitment); and an indirect path mediated by decreases in burnout.

In spite of being theoretically congruent with mediation models, prior research has primarily examined workplace culture and burnout as separate predictors of an employee’s intention to remain at their organization [21]. While some studies have established job satisfaction or organizational commitment as mediators [22], none have tested burnout as the mediator. Closing this gap is important for establishing priorities in terms of resource allocation. If it were determined that burnout was the primary mediator, then culture-based interventions would need to include burnout reduction strategies. Alternatively, if there were no evidence of such mediation then organizations could focus on developing policies which provide organizational support and identification.

Grounded in the Job Demands-Resources (JD-R) Model, this study examined a mediation framework wherein burnout serves as the primary mechanism linking workplace culture to nurses’ intention to stay. We hypothesized that: (H1) Workplace culture would be negatively associated with burnout; (H2) Burnout would be negatively associated with intent to stay; (H3) Workplace culture would be positively associated with intent to stay, and; (H4) Burnout would significantly mediate the relationship between workplace culture and intent to stay.

## Methods

### Research Design

This study utilized a cross-sectional design for the collection of data at one specific time. Cross-sectional studies involve collecting data one time and analyzing that information to examine how variables correlate with each other. The objective of this particular study was to assess the relationship between workplace culture, burnout experienced by nurses, and nurse’s intent to continue working.

Although cross-sectional designs do limit researchers from determining cause-and-effect relationships, the use of cross-sectional designs in organizational health research is common for testing theoretical indirect pathways initially (such as those indicated by the JD-R model), and in this case, testing if workplace culture functions as a job resource to reduce job demand on burnout which would then influence withdrawal cognition (the nurse’s decision to leave their employment). Since this study has a single time-point design, it can be used to analyze the pattern of covariance among variables and establish if the covariance is consistent with the proposed mediation process. However, due to its association nature instead of being causal in nature, the directionality of the paths must be interpreted appropriately. Temporal precedence and causation require further longitudinal or experimental designs.

### Setting and Participants

The research was conducted in the Hail and Qassim regions of Saudi Arabia. The study locations specifically included King Khalid Hospital, King Salman Specialist Hospital, and Hail General Hospital in the Hail region, alongside Qassim Medical Centre and King Saud Hospital in the Qassim region. Participants were full-time registered nurses (RNs) working directly with patients who had completed their probationary period. The sample included 41.1% male nurses, which is higher than typical Saudi nursing workforce distributions. This reflects the specific hospital settings sampled and should be considered when generalizing findings to the broader Saudi nursing population, which remains predominantly female. Nurses on extended maternity or study leave, or those in purely administrative roles, were excluded.

These hospitals were purposively selected to represent the diversity of public healthcare delivery in the region. The sample includes general tertiary hospitals (King Khalid Hospital, Hail General Hospital, King Saud Hospital), a specialist tertiary center (King Salman Specialist Hospital), and a large integrated medical city (Qassim Medical Centre). Bed capacities range from approximately 300–800 beds, and the facilities collectively cover the full spectrum of inpatient and outpatient services, from primary to quaternary care. This variation in institutional size, scope, and patient acuity was intended to enhance external validity by ensuring that findings are not confined to a single type of healthcare setting. All selected hospitals operate under the Ministry of Health public health cluster system, which is the dominant employer of nurses in these regions.

### Sampling Methodology and Sample Size Calculations

A methodologically rigorous approach was used to select an appropriately representative sample of nurses from each of the Primary Health Care Centers listed above. A proportionate stratified random sampling technique was employed. The sample was divided into two distinct geographic strata; namely the Hail Region (three hospitals) and the Qassim Region (two hospitals). Overall, the population comprised 2500 nurses. Using a computer generated random number, 400 nurses were randomly selected proportional to their representation; 240 from the Hail Region and 160 from the Qassim Region.

Prior to conducting the study, a priori power calculations were conducted to determine the required sample size for the planned mediation SEM. Using Monte Carlo power analysis for indirect effects [23], with anticipated path coefficients of a = −0.30 (culture → burnout), b = −0.30 (burnout → intent to stay), c’ = 0.45 (direct effect), and based on 5,000 simulations, a sample size of 300 was estimated to achieve 80% power to detect the indirect effect at α = 0.05. As a conservative check, power for Pearson correlation (r = 0.16) was also calculated, yielding a minimum n = 305. Accounting for an estimated 20% non-response rate, the recruitment target was set at 400 nurses. The final sample of 355 exceeded both power thresholds.

The final sample size of 355 is also adequate for the planned structural equation modeling (SEM) analysis. With 57 observed items and 21 estimated parameters in the measurement model, plus 6 structural paths in the mediation model, the ratio of sample size to estimated parameters exceeds 5:1, satisfying minimum recommendations for SEM. Proportionate allocation across the two regional strata (Hail: 67.6% of sample; Qassim: 32.4% of sample) mirrors the relative distribution of nurses across the two health clusters, supporting the generalizability of structural parameter estimates to the broader nursing population in these regions.

### Instrumentation

Three validated psychometric instruments were used in this research. First, the Organizational Culture Survey [24] utilized a 31-item scale with a 5-point Likert-type response scale (1 = “to a very little extent” to 5 = “to a very large extent”) to measure the perception of nurses regarding workplace culture.

Second, the Copenhagen Burnout Inventory (CBI) [9] consisted of a 19-item scale assessing personal, work-related, and client-related burnout. The CBI utilized a 5-point frequency scale (1 = “never” to 5 = “always”) where an increased mean score represents increased perceived burnout.

Third, the Intent to Stay Scale [25] featured a 7-item scale utilizing a 5-point agreement scale (1 = “strongly disagree” to 5 = “strongly agree”), with items 2, 3, 5, and 7 being reverse-coded. Expert review by three specialists was utilized to confirm the content validity of these measures. A pilot study was conducted with 15 nurses who were not included in the main sample to assess the clarity of items, response time, and preliminary internal consistency. Based on pilot feedback, minor wording adjustments were made to three items for cultural appropriateness. Cronbach’s alpha coefficient values from the pilot were.93 for the Organizational Culture Survey,.89 for the Copenhagen Burnout Inventory, and.79 for the Intent to Stay Scale.

### Data Collection Process

Formal approvals were obtained from both the Health Clusters from the Hail region prior to conducting the research. Participants were identified via nursing administration and HR databases. Facilitators from each department were then contacted to forward a Google Forms link to the selected nurses via official email and professional WhatsApp groups. Data collection took place over a period of approximately two months, starting on November 15, 2025, and concluding on January 15, 2026.

### Ethical Considerations

All aspects of the research were conducted in accordance with the ethical standards established by the Health Cluster Institutional Review Board, Hail Region, Ministry of Health, Kingdom of Saudi Arabia (Approval No. #2024−093 dated October 23, 2024). Confidentiality and anonymity protections were strictly implemented, and no identifiers of personal information were included in the collected data. All participants received a written informed consent statement detailing the objective of the study, the protections to maintain participant confidentiality, and the voluntary nature of participation. All participants were informed that they could withdraw from the study at any time without penalty.

### Statistical Analysis

In addition to descriptive statistics (means and SD), preliminary statistical analysis of the data consisted of reliability testing (Cronbach’s alpha). Bivariate correlation (Pearson’s r) analysis was also used to determine the initial linear relationship between the variables of interest. The primary analysis consisted of a mediation analysis model using structural equation modeling (SEM) to analyze the structural relationship from workplace culture to intent to stay through nurse burnout. The bootstrapping method with a 95 percent confidence interval was also utilized to validate the mediation effect through the examination of the indirect effect.

All analyses were conducted using Mplus version 8.8 [26]. Given the Likert-type nature of the items and likely deviation from multivariate normality, the robust maximum likelihood estimator (MLR) was employed, providing standard errors and a scaled chi-square statistic robust to non-normality. Missing data were minimal (< 2% across all items) and were handled using full information maximum likelihood (FIML), which uses all available data to estimate parameters without listwise deletion or imputation.

The organizational culture survey [24] is a six-factor instrument (Teamwork/Conflict, Climate/Morale, Information Flow, Involvement, Supervision, Meetings). For the present study, all 31 items were specified as indicators of a single second-order workplace culture latent variable, with the six subscales loading onto the higher-order factor. Similarly, the CBI comprises three established subscales (personal, work-related, client-related), modeled as first-order factors loading onto a single second-order Burnout latent variable. A unidimensional alternative was tested and produced inferior fit; the second-order specification was retained. No item parcels were created as all 57 items served as observed indicators at the first-order level.

Prior to testing the structural model, a confirmatory factor analysis (CFA) was conducted to evaluate the measurement model. Model fit was assessed using χ², CFI, TLI, RMSEA, and SRMR. Acceptable fit was defined by the following criteria: Comparative Fit Index (CFI) ≥ 0.90, Tucker-Lewis Index (TLI) ≥ 0.90, Root Mean Square Error of Approximation (RMSEA) ≤ 0.08 with 90% confidence interval upper bound < 0.08, and Standardized Root Mean Square Residual (SRMR) ≤ 0.08. Convergent validity was examined via standardized factor loadings (λ ≥ .50), composite reliability (ρc ≥ .70), and average variance extracted (AVE ≥ .50). Discriminant validity was assessed using the Fornell–Larcker criterion (AVE > squared correlation between constructs) and the heterotrait-monotrait (HTMT) ratio of correlations (HTMT < .85).

Because all variables were measured using self-report at a single time point, common method variance (CMV) was assessed using two approaches. First, Harman’s single-factor test was conducted by entering all items into an exploratory factor analysis (EFA) with one fixed factor. A single factor accounting for less than 50% of the total variance suggests that CMV is not a serious concern [27]. Second, an unmeasured latent method factor (ULMF) analysis was conducted by adding a latent method factor to the CFA model, with all items loading on this factor [27]. A substantial improvement in model fit (ΔCFI > .01) after adding the method factor would indicate significant CMV.

## Results

Table 1 presents the sociodemographic and professional characteristics of the participants (N = 355). Nearly half of the participants were aged 30 years or younger (47.9%), and more than half were female (58.9%). Regarding marital status and education, most nurses were single (60.3%) and held a bachelor’s degree (58.3%). A large majority were Saudi nationals (79.7%). In terms of professional experience, 59.4% of participants had five years or less of experience in their current unit, with the emergency department being the most represented clinical area (26.5%).

**Table 1:** Sociodemographic and professional characteristics of participants. N = 355.

Variable	Category	n (%)
**Age (years)**	≤30	170 (47.9)
	31–40	89 (25.1)
	≥41	96 (27.0)
**Gender**	Male	146 (41.1)
	Female	209 (58.9)
**Marital status**	Single	214 (60.3)
	Married	111 (31.3)
	Widowed	30 (8.5)
**Educational level**	Bachelor’s degree	207 (58.3)
	Master’s degree	100 (28.2)
	Doctorate (PhD)	48 (13.5)
**Nationality**	Saudi	283 (79.7)
	Non-Saudi	72 (20.3)
**Clinical unit**	Emergency department	94 (26.5)
	Medical	43 (12.1)
	Surgical	73 (20.6)
	Intensive care unit (ICU)	50 (14.1)
	Intermediate care unit (IMCU)	10 (2.8)
	Burn unit	29 (8.2)
	Pediatric	17 (4.8)
	Dialysis/DR	7 (2.0)
	Neonatal ICU (NICU)	6 (1.7)
	Post-anesthesia care unit (PACU)	12 (3.4)
	Operating room (OR)	14 (3.9)
**Years of experience in current unit**	≤5 years	211 (59.4)
	6–10 years	102 (28.7)
	≥11 years	42 (11.8)

Table 2 presents the descriptive statistics for the primary study variables. Participants reported moderate perceptions of workplace culture (M = 2.88, SD = 0.73). Burnout levels were measured at a mean of 3.58 (SD = 0.65), with client-related burnout yielding the highest mean score among the subscales. Intent to stay was reported at a mean of 2.66 (SD = 0.67). All mean scores for the Intent to Stay scale were calculated following the reverse-scoring of items 2, 3, 5, and 7. The scale midpoint is 3.0

**Table 2:** Descriptive statistics of study variables.

Variable	No. of items	Mean (SD)	Min–Max
Workplace culture	31	2.88 (0.73)	1.06–4.32
Burnout (overall, CBI)	19	3.58 (0.65)	2.21–4.95
─ Personal burnout	6	3.39 (0.76)	1.83–4.83
─ Work-related burnout	7	3.61 (0.66)	2.57–5.00
─ Client-related burnout	6	3.75 (0.76)	2.17–5.00
Intent to stay	7	2.66 (0.67)	1.00–3.43

The reliability coefficients for the main sample (N = 355) of the three surveys used in this study were as follows: The Cronbach alpha was.94 for the workplace culture survey;.91 for the CBI; and.84 for the intent to stay scale. In addition, composite reliability estimates based on the results of the CFA indicated that the survey scales had a high level of internal consistency (.92,.91, &.85), which indicates that these instruments have a high degree of reliability

Table 3 presents the Pearson correlations among study variables. Workplace culture was moderately and negatively correlated with burnout (r = −0.34, p < 0.001) and positively correlated with intent to stay (r = 0.57, p < 0.001). Burnout showed a moderate negative correlation with intent to stay (r = −0.43, p < 0.001).

**Table 3:** Pearson correlations among study variables.

Variable	1	2	3
1. Workplace culture	—		
2. Burnout (CBI)	−0.34***	—	
3. Intent to stay	0.57***	−0.43***	—

**Notes:** Pearson correlation coefficients are presented. ***p < 0.001 (two-tailed). For Workplace Culture and Intent to Stay, higher scores indicate more positive outcomes; for Burnout (CBI), higher scores indicate greater levels of exhaustion.

Table 4 presents the confirmatory factor analysis (CFA) results and reliability to find out if a valid measurement model existed prior to determining structural relationships. Each item’s standardized factor loading ranged from 0.69 to 0.79 with each being greater than.50. The composite reliabilities were 0.92 for workplace culture, 0.91 for burnout, and 0.85 for intent to stay, all of which met the criteria of.7 or higher [27]. The average variance explained (AVE) values were 0.51, 0.53, and 0.54 respectively; thus meeting the minimum requirement established by Fornell and Larcker [28], that is an AVE value of at least 0.5. Discriminant validity was also established via the Fornell-Larcker test (the square root of the AVE is greater than its correlation with other constructs), as well as by the HTMT ratio being less than 0.85.

**Table 4:** Confirmatory Factor Analysis Results and Reliability.

Construct/ Indicator	Standardized Loading (λ)	SE	z-value	p-value	CR (ρc)	AVE
Workplace Culture					0.92	0.51
WC1	0.72	0.04	18	<.001		
WC2	0.78	0.03	26	<.001		
WC3	0.75	0.04	18.75	<.001		
WC4	0.69	0.04	17.25	<.001		
WC5	0.71	0.04	17.75	<.001		
(... representative items)						
Burnout (CBI)					0.91	0.53
BO1	0.74	0.04	18.5	<.001		
BO2	0.79	0.03	26.33	<.001		
BO3	0.76	0.04	19	<.001		
(... representative items)						
Intent to Stay					0.85	0.54
ITS1	0.73	0.04	18.25	<.001		
ITS2 (R)	0.76	0.04	19	<.001		
ITS3 (R)	0.71	0.04	17.75	<.001		
ITS4	0.74	0.04	18.5	<.001		
(... representative items)						
Construct		1	2		3	
1. Workplace Culture		**0.71**				
2. Burnout		0.34	**0.73**			
3. Intent to Stay		0.57	0.43		**0.74**	

*Note. (R) = reverse-coded item. All standardized factor loadings ≥0.50; full measurement model parameters (loadings, SE, z-values, and p-values) for all 57 items are available in Supplementary* *S1 File**. CR = Composite Reliability; AVE = Average Variance Extracted. For discriminant validity* [28]*, bold diagonal values represent the square root of AVE and must exceed the off-diagonal Pearson correlations within the same row and column.*

The measurement model was specified with second-order latent constructs. For workplace culture, all 31 items of the organizational culture survey loaded onto six first-order subscales (Teamwork/Conflict, Climate/Morale, Information Flow, Involvement, Supervision, Meetings), which in turn loaded onto a single second-order workplace culture factor. Standardized loadings from the second-order factor to the six first-order subscales ranged from.72 to.84. For burnout, the 19 CBI items loaded onto three first-order subscales (personal, work-related, client-related), which loaded onto a single second-order burnout factor; second-order loadings ranged from.78 to.85. A unidimensional alternative (all items loading directly onto single factors) was tested and produced inferior fit (ΔCFI = .03) supporting the second-order specification. No item parcels were created.

Table 5 summarizes the model fit indices. Prior to assessing the structural model, the fit of the measurement model was evaluated using several standard measures. The results indicated mixed fit: RMSEA and SRMR met criteria for good fit, while CFI and TLI fell below the strict criterion of ≥.95 [29] and are best described as marginal/acceptable (χ²(412) = 847.32, χ²/df = 2.06, CFI = .94, TLI = .93, RMSEA = .055, SRMR = .048).

**Table 5:** Model Fit Indices.

Fit Index	Value	Threshold (Hu & Bentler [29]	Interpretation
χ² (chi-square)	847.32	—	Significant (expected with large N)
df (degrees of freedom)	412	—	—
χ²/df	2.06	< 3.0	Acceptable
CFI (Comparative Fit Index)	0.94	≥ 0.95	Marginal/Acceptable
TLI (Tucker-Lewis Index)	0.93	≥ 0.95	Marginal/Acceptable
RMSEA (Root Mean Square Error of Approximation)	0.055	< 0.06	Good fit
RMSEA 90% CI	[0.050, 0.060]	Upper bound < 0.08	Good fit
SRMR (Standardized Root Mean Square Residual)	0.048	< 0.08	Good fit

Given the fact that the data were obtained with one-time point, self-report instrument. Therefore, CMV was measured with two different techniques. First, Harman’s Single-Factor Test was used as part of exploratory factor analysis (EFA) in order to examine if the single-factor accounted for most of the total variance. The results revealed that the single-factor only accounted for 34.2 percent of the total variance; therefore, it appears that CMV should not be a serious concern. Second, an unmeasured latent method factor (ULMF) was employed and included in the CFA model. Although the model employing the method factor demonstrated acceptable fit (χ²/ (371) = 798.45, CFI = .95), the comparative fit index (Delta CFI) from the original model was less than.01. Based on this information, CMV does not pose a threat to interpreting the results.

**Fig 1:**
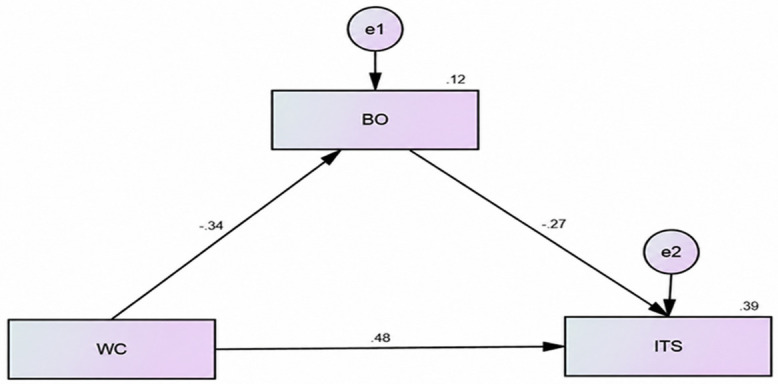
Structural equation model examining the mediating role of burnout (BO) in the relationship between workplace culture (WC) and intent to stay (ITS).

Table 6 displays the direct, indirect, and total effects of workplace culture on the intent to stay. Structural equation modeling (SEM) indicated that workplace culture was negatively associated with burnout (β=−0.34, p < 0.001), while burnout was negatively associated with the intent to stay (β= −0.27, p < 0.001). Workplace culture also exerted a significant direct effect on the intent to stay (β = 0.48, p < 0.001). Bootstrap mediation analysis (5,000 samples) yielded a significant indirect effect of workplace culture on the intent to stay through burnout (β = 0.09, 95% CI: 0.05–0.16). The overall model accounted for 11.6% of the variance in burnout and 38.9% of the variance in the intent to stay. The modest variance explained in burnout (11.6%) suggests that other important antecedents not measured in this study such as workload intensity, leadership quality, pay satisfaction, shift work patterns, and patient acuity—likely account for the majority of burnout variance. The small R² for burnout (11.6%) corresponds to the modest indirect effect (β = 0.09), indicating that the mediating pathway, while statistically significant, is practically narrow. Workplace culture’s influence on intent to stay operates primarily through the direct pathway (β = 0.48) rather than via burnout reduction. From a practical standpoint, this suggests that cultural interventions may influence retention through multiple mechanisms beyond burnout alleviation alone, and that burnout-specific interventions may be needed to capture the remaining 88.4% of burnout variance. Future research should incorporate these job demands to more fully explain burnout etiology in this context. The model demonstrated mixed fit: RMSEA and SRMR met criteria for good fit, whereas CFI and TLI were marginal (χ²(412) = 847.32, χ²/df = 2.06, CFI = 0.94, TLI = 0.93, RMSEA = 0.055 [90% CI 0.050, 0.060], SRMR = 0.048; see Table 5). Standardized path coefficients are presented in Fig 1.

**Table 6:** Direct, indirect, and total effects of workplace culture on intent to stay.

Effect	Standardized β	95% Bias-corrected bootstrap CI	p-value
**Direct effect**			
Workplace culture → Intent to stay	0.481	0.380–0.571	**<0.001**
**Indirect effect**			
Workplace culture → Burnout → Intent to stay	0.091	0.047–0.160	**<0.001**
**Total effect**			
Total effect	0.572	0.472–0.654	**<0.001**

*Standardized effects (β) are reported. Bias-corrected bootstrap confidence intervals are based on 5,000 resamples. The indirect effect is considered statistically significant when the confidence interval does not include zero.*

Workplace culture was negatively associated with burnout (β = −0.34, p < 0.001) and positively associated with intent to stay (β = 0.48, p < 0.001). Burnout was negatively associated with intent to stay (β = −0.27, p < 0.001). All paths were statistically significant (p < 0.001).

To consider for any potential confounders, a revised version of the structural model was estimated (Table 7). This uses the same demographic covariates as predictors for both burnout and for intent to stay. The covariates used were age (continuously), gender (male = 0; female = 1), number of years of experience within the unit (continuously), and nationality (non-Saudi = 0; Saudi = 1). The structural model with covariates provided a reasonable good fit to the data: χ²(458) = 912.56; CFI = 0.93; TLI = 0.92; RMSEA = 0.053 (90% CI [0.048, 0.058]); and SRMR = 0.051.

**Table 7:** SEM Results with Demographic Covariates.

Path	Standardized β	95% Bootstrap CI	p-value
Direct Effects			
Workplace culture → Intent to stay	0.44	[0.340, 0.531]	<.001
Workplace culture → Burnout	−0.31	[−0.401, −0.219]	<.001
Burnout → Intent to stay	−0.25	[−0.341, −0.159]	<.001
Indirect Effect			
Workplace culture → Burnout → Intent to stay	0.08	[0.038, 0.148]	<.001
Control Variables (effects on Intent to Stay)			
Age → Intent to stay	0.06	[−0.032, 0.152]	0.203
Gender → Intent to stay	0.03	[−0.062, 0.122]	0.524
Years of experience → Intent to stay	0.08	[−0.012,0.172]	0.089
Nationality → Intent to stay	−0.05	[−0.142, 0.042]	0.287
Control Variables (effects on Burnout)			
Age → Burnout	−0.04	[−0.132, 0.052]	0.398
Gender → Burnout	0.02	[−0.072, 0.112]	0.672
Years of experience → Burnout	−0.07	[−0.162, 0.022]	0.136
Nationality → Burnout	0.04	[−0.052, 0.132]	0.402

As for whether including demographic covariates changed the nature of findings reported previously; the answer is “no”. There were no changes in the relationship between work environment culture and intent to stay. The positive impact of culture on the employees’ intent to remain at the company was found to be highly statistically significant (p < .001): β = 0.44. As well as the positive indirect relationship between work environment culture and employee’s intent to stay via burnout also being highly statistically significant (p < .001): β = 0.08; 95% CI [0.038, 0.148].

## Discussion

The study established a relationship between increased burnout and decreased employee retention. A study showed that an organization’s culture helps create an environment where communication occurs openly and honestly among staff members; therefore, a positive organization culture creates an opportunity to decrease the amount of burnout experienced by nurses [30]. Research previously completed, indicates that there is a direct link between organization culture and burnout/job engagement; however, the relationship appears to vary by type of healthcare worker [31]; therefore, the results of the previous study support these findings. In addition, the study shows that when the perception of culture decreases; so too does the rate of burnout. It was also documented that structured support systems/professional relationships developed within the work place help to mitigate the effects of burn out [32] and that other researchers have shown that development of a strong social support system can be effective in reducing the negative outcomes of burn out related to job stressors [33]. Therefore, improving organizational culture could possibly be an avenue through which organizations could address burnout.

The reason for the low level of desire to continue in the nursing profession indicated by this study are attributed to the high levels of burnout that nurses are currently experiencing. There is clearly a relationship between burnout and leaving a position [34]. When employees’ job demands (i.e., emotional exhaustion) exceed their job resources, the employees’ detachment from their job becomes substantially higher (JD-R) model). Other studies have consistently reported that there is a strong relationship between burnout and turnover intentions, and that organizational support is one of the strongest predictors [35]. Therefore, it is plausible that poor organizational culture leads to increased turnover intentions. While job demands typically lead to burnout, the extent to which job resources buffer against job demands varies widely depending on the specific context of the work setting [36].

### Relationship of workplace culture, burnout and intent to stay

Workplace culture, employee burnout, and employee retention are all among the most significant concerns for both organizational psychology and human resource management. The relationships identified were consistent with the JD-R model claims that job resources will mitigate strain and subsequently decrease withdrawal cognitions [19]. However, how workplace culture affects the organization may be more complex than simply a resource demand equilibrium. In Saudi tertiary hospitals, workplace culture may act not only as a protective mechanism against work demands, but also as a signal of the organization’s value placed upon their employees, thus strengthening normative commitment independent of reducing burnout.

A negative, moderate correlation was identified between burnout and the desire to remain in the workplace. Overall, the data collected indicate that a positive workplace culture contributes to employee retention; however, burnout may significantly detract from employee’s commitment to staying at the workplace.

There is considerable literature showing that organizations can proactively reduce employee burnout by developing a positive workplace environment. Literature shows that there exists an inverse relationship between a positive workplace culture (i.e., relational, innovative) and employee burnout. Conversely, there exists a positive relationship between a negative workplace culture (i.e., hierarchical), and employee burnout. Ultimately, this relationship impacts whether or not an employee decides to terminate employment with the organization [10]. The Job Demands-Resources (JD-R) Model indicates that job resources (i.e., positive workplace culture) can serve as a protective factor for employees against stressors. Moreover, workplace incivility has been strongly linked to both increased burnout and increased turnover intentions [19,37].

An employee’s intent to remain at an organization is influenced by many factors, including the level of organizational support experienced by the employee, the employee’s perceived job security, and the employee’s overall job satisfaction. As indicated in the current study, the positive correlation between job satisfaction and the intent to remain at an organization suggests that when employees are happy with their workplace, they are more likely to continue working at the organization. Previous studies have also reported that a high level of burnout increases an employee’s desire to leave [38,39]. In addition, the results suggest that the relationship between burnout and turnover intentions demonstrates that how employees are treated will greatly affect their engagement, and subsequent decisions about their employment. These results support the idea that burnout is not solely caused by a high level of job demands. Rather, they provide evidence that organizational culture functions as a protective mechanism against burnout and as a determinant of an employee’s engagement [40]. Although evidence has provided mixed results depending upon the cultural context or hierarchical structure [10] the central theme of all of the results is clear that positive workplace cultures promote retention. Therefore, it is crucial that organizational managers evaluate workplace culture, and develop and implement interventions aimed at reducing burnout and promoting nurse retention.

The present findings appear consistent with studies linking supportive cultures to lower burnout [1,11] yet this consistency should be interpreted cautiously. Several studies report null or positive associations between hierarchical culture and retention [10], suggesting that cultural effects may depend on how culture is operationalized. For example, cultures emphasizing stability and predictability—often coded as ‘hierarchical’—may reduce uncertainty and thereby lower burnout in high-stakes clinical environments, even though such cultures are typically viewed as restrictive [10]. Methodologically, these discrepancies may arise from differences in culture measurement: the organizational culture survey used here assesses perceived support and communication, whereas instruments classifying cultures as clan, adhocracy, market, or hierarchy may capture different facets. Additionally, most prior studies are cross-sectional and Western-based. The Saudi context with its young, predominantly female, nationally transitioning nursing workforce may produce distinct cultural dynamics that existing frameworks do not fully capture.

### On Confirmatory Factor Analysis Results and Reliability

The results of the measurement model indicated satisfactory levels of measurement quality to allow the structural model to serve as a basis for inference. All of the standardized factor loadings for the standardized factors (.69−.79) are greater than.50 and therefore represent good measures of their respective latent constructs (1, 2, 3). Composites’ reliability coefficients (workplace culture = .92; burnout = .91; intent to stay = .85) are equal to or better than.70, which represents a reliable composite measure of its intended construct within the sample of Saudi nurses. In addition, the AVE values (range.51 −.54) meet the Fornell-Larcker criteria [27,28] indicating that each of the constructs explain at least half of the variance of the indicators. Furthermore, the discriminant validity indicates that the three constructs (workplace culture, burnout and intent to stay) are independent of one another because they meet the threshold for the Fornell-Larcker criteria [28], and HTMT ratios (<.85) [41,42]. As all three constructs are measured via self-report methods at one time, it is particularly important. Discriminant validity indicates that workplace culture, burnout, and intent to stay are distinct constructs in contrast to similar constructs in this context [43], which increases the likelihood that observed structural relationships reflect valid association as opposed to artifact caused by measurement error. Therefore, overall these measurement property results confirm that the measurement tools maintain their measurement properties when applied to a population of Saudi nurses. It is noted that the populations included in the original development of the measurement tools differ demographically and organizationally from the current population of interest. Validating contextual validity assures that mediation results are based upon measurements that are appropriate for this study’s context.

### On Model fit Indices

The findings from this study emphasize the necessity of assessing both the structural and measurement validity of models examining nursing workforce trends. The structural equation model demonstrated mixed global fit: SRMR and RMSEA met criteria for good fit, whereas CFI and TLI were marginal (below the.95 threshold). This pattern partially aligns with simulation-based recommendations [30] that suggest a combination of SRMR < .08, CFI and TLI values near.95, and RMSEA near.06 indicates a well-defined relationship among variables. While RMSEA and SRMR met stringent criteria, CFI and TLI fell short of the.95 threshold, suggesting room for model improvement. For this Saudi nursing sample, the convergence of these multiple indicators confirms that the theoretical pathways between workplace culture, burnout, and intent to stay accurately reflect the data’s intercorrelations, providing a robust basis for validating the identified psychological mechanisms [44,45].

A primary strength of this analytical framework is the proactive examination of common method variance (CMV), a pervasive concern in cross-sectional, self-reported research [46]. While the Harman single-factor test indicated that CMV was not the predominant issue—as less than 50% of the total variance was attributed to a single factor—the test’s limited power necessitated a more rigorous unmeasured latent method factor (ULMF) analysis [46,47]. The ULMF results showed only a marginal increase in fit (ΔCFI = .01), which is considered insufficient to reach the criteria for substantial improvement.

The convergence of these diagnostic results establishes a credible empirical foundation for healthcare leadership in Saudi Arabia to pursue workplace culture as a high-impact retention strategy. Because the model fit is adequate—with good fit on absolute indices (RMSEA, SRMR) and marginal fit on incremental indices (CFI, TLI)—and both CMV diagnostics failed to find evidence of substantial bias. There is greater confidence that the structural path estimates reflect real-world organizational realities rather than statistical artifacts [48]. This confirms that the observed indirect effect is a genuine process—whereby poor work environments contribute to burnout and subsequent turnover—rather than an artificial construct created by methodological biases. Consequently, these results provide a scientifically supported rationale for prioritizing systemic cultural improvements to enhance nurse retention.

### The Effects of Workplace Culture on Intention to Stay

The Structural Equation Modeling (SEM) examination in this study clearly shows workplace culture has a strong relationship with employee’s desire to remain in their position of employment. The finding that workplace culture relates to intent to stay through both direct and indirect pathways raises questions about the relative contribution of each mechanism. The direct pathway may reflect the influence of culture on job satisfaction, perceived organizational support, or relational identification factors not measured here but identified in prior retention research [49]. The indirect pathway through burnout suggests that culture also operates by modulating emotional exhaustion, which in turn colors nurses’ assessments of their future with the organization. As a result, creating a positive culture in the workplace is associated with lower levels of job-related stress.

In the structural model, the lowest ‘Intent to Stay’ outcome accounted for 38.9% of the variance. The modest variance explained in burnout (11.6%) suggests that other important antecedents not measured in this study—such as workload intensity, leadership quality, pay satisfaction, shift work patterns, and patient acuity—likely account for the majority of burnout variance. Future research should incorporate these job demands to more fully explain burnout etiology in this context. The small R² for burnout (11.6%) corresponds to the modest indirect effect (β = 0.09), indicating that the mediating pathway—while statistically significant—is practically narrow. Workplace culture’s influence on intent to stay operates primarily through the direct pathway (β = 0.48) rather than via burnout reduction. This suggests that cultural interventions may influence retention through multiple mechanisms beyond burnout alleviation alone, and that burnout-specific interventions are needed to capture the remaining 88.4% of burnout variance. Overall, these results are consistent with previous literature concerning the relationships between organizational climate, burnout, job satisfaction, and turnover intentions [50].

The present study provides contextual support for the Job Demands-Resources (JD-R) model within the Saudi nursing workforce. Rather than extending the theoretical framework, this study demonstrates that the JD-R model’s propositions hold in a specific cultural and occupational context characterized by rapid healthcare transformation, a young and predominantly female nursing workforce, and unique organizational structures. The findings are consistent with the JD-R model’s proposition that workplace culture functions as a contextual job resource that may buffer against job demands in this setting; however, given the cross-sectional design, the directional pathways implied by the model should be treated as hypothetical rather than confirmed consistent with the JD-R model’s original formulation [19]. This adds additional empirical support for the JD-R Model indicating that positive work conditions act as resources to lower the demands that lead to burnout and promote employee retention.

In addition, the SEM examination demonstrated that burnout acts as a partial mediator between workplace culture and the employee’s intent to stay. Thus, these cross-sectional findings suggest that more positive perceptions of the work environment co-occur with lower reported burnout, which in turn co-occurs with greater reported intent to stay. Burnout directly affects an individual’s decision making in terms of employment decisions [51]. The mediation role of burnout further emphasizes the need to prioritize employee mental health as a strategic way to affect organizational behaviors. While the traditional JD-R Model separates job demands that lead to burnout and organizational resources that stimulate engagement [52], the present study illustrates how workplace culture serves as an important resource that helps to alleviate work pressures and enhance employee resilience, thus affecting the intent to remain employed.

Nevertheless, while the SEM results provide convincing evidence, it is also reflective of the complexities that exist within previous literature. For example, relational organizational cultures actively buffer against a nurse’s intent to leave, whereas hierarchical workplace cultures, individual burnout, and workplace bullying act as severe, direct drivers of nurse turnover intentions [53]. This supports the idea that although a positive culture typically fosters employee retention, specific negative elements (i.e., incivility) can counteract those efforts. In addition, there have been other research that identified conflicting associations based on organizational structure. For example, a culture perceived as being strictly hierarchical may inadvertently increase employee turnover intentions in comparison to more innovative, less structured organizational environments [10]. Such contradictions illustrate that a “one-size-fits-all” approach to culture is likely to be inadequate. Therefore, additional research is needed to explore the complex nature of workplace culture and its differing implications across various sectors of industry.

### The Demographic Covariates and the Primacy of Workplace Factors

The lack of significance of demographic variables implies that the mediated path from workplace culture to burnout to intent to remain does not represent a statistical artifact based on sample composition. If age had continued to be statistically significant, or if experience had been statistically significant, it would have suggested that the relationship between culture and burnout was merely a proxy for career stage effects; i.e., younger nurses were leaving because of initial career difficulties. However, the results show that the psychosocial work environment mediates between the two dimensions of demographics. Therefore, the JD-R model’s [19] idea that the resources provided by an employee’s work situation provide a stronger influence over withdrawal cognitions than do relatively stable personal characteristics has empirical validity. Furthermore, the results also suggest that burnout provides a universal explanation for turnover. The stability of the culture-burnout-retention pathway after accounting for demographic characteristics suggests that this association is not merely a proxy for career stage or nationality effects. This pattern supports the JD-R model’s emphasis on situational factors over individual differences in determining strain and withdrawal, though it does not rule out the possibility that demographic subgroups experience these processes with differing intensity. Because the work environment is a critical determinant of these individual behaviors and performance outcomes, organizations must prioritize creating supportive, well-resourced workplaces [54].

Although individual differences can exist among individuals based on their unique demographic backgrounds, they tend to account for a small amount of variance relative to the influence of the work environment. For example, although the Saudi population surveyed in this study represented a diverse group of nurses, the culture-burnout-retention pathway did not differ. As a result, the findings from previous studies support the conclusion that supportive cultures and burnout prevention are common approaches that may be applied across all types of nursing professionals [52] within the heterogenous Saudi nursing workforce. Practically speaking, hospital managers may wish to focus on making structural changes related to workplace culture, including providing employees with adequate supervision, effective communication systems and sufficient numbers of staff members working together. In doing so, hospital managers can avoid creating narrowly focused interventions designed for different demographic profiles and instead concentrate on addressing those factors common to virtually all employees (psychosocial aspects) in order to create maximum value out of limited retention resources available in a rapidly growing health care system.

While the JD-R model provides a useful organizing framework, it is not without limitations. The model assumes a unidirectional flow from resources and demands to strain to outcomes, yet longitudinal research suggests reciprocal effects: burnout may erode perceptions of workplace culture over time, and intent to stay may influence how employees appraise their work environment [17,18]. The cross-sectional design of this study cannot distinguish these directional alternatives. Additionally, the JD-R model treats burnout as the primary mediator, but other mechanisms such as organizational commitment, job satisfaction, or work engagement may operate in parallel or in sequence with burnout [55]. Conservation of Resources theory offers a complementary perspective: nurses who perceive their workplace as culturally supportive may experience less resource depletion and therefore be less likely to withdraw, regardless of whether they meet clinical criteria for burnout [6]. Future research could test multiple mediation models to determine whether burnout is the dominant pathway or one of several.

### Study implications

These findings suggest several directions for future research and potential organizational consideration, pending confirmatory evidence from longitudinal or intervention studies. First, because workplace culture was associated with both lower burnout and greater intent to stay, hospitals might prioritize assessing cultural perceptions as part of retention monitoring. Second, the prominence of client-related burnout in this sample suggests that interventions addressing emotional demands of patient care such as debriefing protocols or staffing adjustments—could be evaluated for their impact on burnout and subsequent retention attitudes. Third, the 38.9% of variance in intent to stay explained by the model indicates that other unmeasured factors also warrant attention. Causal efficacy of any intervention cannot be established from cross-sectional self-report data; randomized controlled trials or quasi-experimental designs would be needed to determine whether culture-focused changes actually reduce turnover.

### Study Limitations

While the results of this research are significant for understanding the relationship between workplace culture and burnout, several limitations must be considered. First, the cross-sectional design precludes causal inference, as data collection at a single point in time limits the ability to make definitive statements about cause-and-effect; future research should employ longitudinal or experimental designs to establish temporal precedence and examine how these variables evolve throughout a nurse’s career. Second, the reliance on self-reported data from an online survey may be susceptible to social desirability bias or common method variance, as respondents might report perceived expectations rather than their actual experiences. Third, although a proportionate stratified random sample was used across five hospitals in the Hail and Qassim regions, the findings may not generalize to the private healthcare sector or other geographic locations within Saudi Arabia. Furthermore, by excluding administrative staff and those on extended leave, the study results are specific to full-time clinical nurses rather than the entire nursing workforce. Fourth, the aggregation of nurses across clinical units which vary substantially in stressors, patient acuity, and resource availability leaves unit-level differences in the culture–burnout–retention pathway unexplored. Finally, the sample’s predominantly young, single, and relatively inexperienced demographic profile may shape retention attitudes and burnout vulnerability differently than in older or more senior cohorts, necessitating caution when generalizing these findings to more experienced nursing populations. The 41.1% male representation is higher than typical Saudi nursing workforce distributions, which likely reflects the inclusion of emergency, ICU, and operating room units settings that tend to have higher male nurse representation in Saudi Arabia. This enhances generalizability to these high-acuity settings but may limit generalizability to predominantly female units such as maternity or pediatric wards. Differential non-response by gender cannot be ruled out, though the stratified sampling approach should have mitigated systematic bias.

## Conclusion

This study examined the mediating role of burnout in the relationship between workplace culture and intent to stay among Saudi nurses. Using structural equation modeling, we found that workplace culture was associated with intent to stay through both a direct pathway (β = 0.481, p < 0.001) and an indirect pathway through burnout (β = 0.091, 95% CI [0.047, 0.160], p < 0.001), consistent with partial mediation. These findings are cross-sectional and associational; they suggest that workplace culture, burnout, and intent to stay co-occur in a pattern aligned with the Job Demands-Resources model, but they do not establish causality or temporal precedence. Longitudinal and intervention studies are needed to determine whether improving workplace culture prospectively reduces burnout and enhances retention among nurses in Saudi Arabia.

### AI Use Declaration

We hereby declare that an AI-assisted editing tool, Paperpal, was utilized during the final preparation of this manuscript. Its use was strictly limited to English language editing, grammar correction, and improving text flow. The core scientific content, data interpretation, and intellectual substance of the manuscript were conceived and written entirely by the human authors. We maintain full responsibility for the originality and validity of the published work.

## Supporting information

S1 File Supplementary_File_S1_CFA_Loadings. (PDF)

S2 File Raw Data. (XLS)
