# Supplementary material for: The mediating effect of burnout on the relationship between workplace culture and intent to stay among registered nurses in Saudi Arabia
Source: PLoS One. 2026 Jun 25;21(6):e0352383. doi: 10.1371/journal.pone.0352383 (PMC13298968; doi:10.1371/journal.pone.0352383)
Supplement: S1 File — (PDF) [file pone.0352383.s001.pdf]

Construct	Subscale	Item Code	Item Descr	Reverse Cc	Î»	SE	z	p
Workplace	Teamwork	WC1	Team men	No	0.72	0.04	18	<.001
Workplace	Teamwork	WC6	Conflicts b	No	0.74	0.03	24.67	<.001
Workplace	Teamwork	WC7	There is a	No	0.7	0.03	23.33	<.001
Workplace	Teamwork	WC8	Team men	No	0.76	0.04	19	<.001
Workplace	Teamwork	WC9	Disagreem	No	0.71	0.04	17.75	<.001
Workplace	Teamwork	WC10	Collaborat	No	0.73	0.04	18.25	<.001
Workplace	Climate/M	WC2	The moral	No	0.78	0.03	26	<.001
Workplace	Climate/M	WC11	Staff feel v	No	0.75	0.05	15	<.001
Workplace	Climate/M	WC12	The work e	No	0.77	0.04	19.25	<.001
Workplace	Climate/M	WC13	Employees	No	0.69	0.05	13.8	<.001
Workplace	Climate/M	WC14	There is a	No	0.74	0.03	24.67	<.001
Workplace	Climate/M	WC15	Staff feel t	No	0.71	0.05	14.2	<.001
Workplace	Informatio	WC3	Informatio	No	0.75	0.04	18.75	<.001
Workplace	Informatio	WC16	Important	No	0.73	0.04	18.25	<.001
Workplace	Informatio	WC17	Communic	No	0.7	0.03	23.33	<.001
Workplace	Informatio	WC18	Staff are k	No	0.76	0.03	25.33	<.001
Workplace	Informatio	WC19	There are	No	0.72	0.03	24	<.001
Workplace	Involveme	WC4	Employees	No	0.69	0.04	17.25	<.001
Workplace	Involveme	WC20	Staff have	No	0.71	0.05	14.2	<.001
Workplace	Involveme	WC21	Employees	No	0.74	0.04	18.5	<.001
Workplace	Involveme	WC22	Staff input	No	0.7	0.03	23.33	<.001
Workplace	Involveme	WC23	There are	No	0.72	0.03	24	<.001
Workplace	Supervisio	WC5	Supervisor	No	0.71	0.04	17.75	<.001
Workplace	Supervisio	WC24	Supervisor	No	0.73	0.03	24.33	<.001
Workplace	Supervisio	WC25	Leadership	No	0.69	0.05	13.8	<.001
Workplace	Supervisio	WC26	Supervisor	No	0.75	0.05	15	<.001
Workplace	Supervisio	WC27	Managem	No	0.7	0.04	17.5	<.001
Workplace	Meetings	WC28	Meetings a	No	0.72	0.05	14.4	<.001
Workplace	Meetings	WC29	Staff have	No	0.74	0.03	24.67	<.001
Workplace	Meetings	WC30	Meeting o	No	0.7	0.04	17.5	<.001
Workplace	Meetings	WC31	Decisions r	No	0.73	0.03	24.33	<.001
Burnout (C	Personal B	BO1	How often	No	0.74	0.04	18.5	<.001
Burnout (C	Personal B	BO4	How often	No	0.72	0.03	24	<.001
Burnout (C	Personal B	BO5	How often	No	0.76	0.05	15.2	<.001
Burnout (C	Personal B	BO6	How often	No	0.7	0.04	17.5	<.001
Burnout (C	Personal B	BO7	How often	No	0.73	0.05	14.6	<.001
Burnout (C	Personal B	BO8	How often	No	0.71	0.05	14.2	<.001
Burnout (C	Work-relat	BO2	How often	No	0.79	0.03	26.33	<.001
Burnout (C	Work-relat	BO9	How often	No	0.75	0.04	18.75	<.001
Burnout (C	Work-relat	BO10	How often	No	0.77	0.03	25.67	<.001
Burnout (C	Work-relat	BO11	How often	No	0.73	0.03	24.33	<.001
Burnout (C	Work-relat	BO12	How often	No	0.7	0.04	17.5	<.001
Burnout (C	Work-relat	BO13	How often	No	0.74	0.03	24.67	<.001
Burnout (C	Work-relat	BO14	How often	No	0.72	0.04	18	<.001
Burnout (C	Client-rela	BO3	How often	No	0.76	0.04	19	<.001
Burnout (C	Client-rela	BO15	How often	No	0.78	0.04	19.5	<.001
Burnout (C	Client-rela	BO16	How often	No	0.71	0.05	14.2	<.001
Burnout (C	Client-rela	BO17	How often	No	0.75	0.04	18.75	<.001
Burnout (C	Client-rela	BO18	How often	No	0.73	0.05	14.6	<.001

Burnout (C	Client-rela	BO19	How often	No	0.7	0.03	23.33	<.001
Intent to S	Intent to S	ITS1	I intend to	No	0.73	0.04	18.25	<.001
Intent to S	Intent to S	ITS2	I often thir	Yes	0.76	0.04	19	<.001
Intent to S	Intent to S	ITS3	I would ac	Yes	0.71	0.04	17.75	<.001
Intent to S	Intent to S	ITS4	I plan to m	No	0.74	0.04	18.5	<.001
Intent to S	Intent to S	ITS5	If I could cl	Yes	0.72	0.03	24	<.001
Intent to S	Intent to S	ITS6	I feel loyal	No	0.7	0.03	23.33	<.001
Intent to S	Intent to S	ITS7	I would lea	Yes	0.75	0.03	25	<.001
